# Locomotor rib kinematics in two species of lizards and a new hypothesis for the evolution of aspiration breathing in amniotes

**DOI:** 10.1038/s41598-020-64140-y

**Published:** 2020-05-12

**Authors:** Robert L. Cieri, Samuel T. Hatch, John G. Capano, Elizabeth L. Brainerd

**Affiliations:** 1 School of Biological Sciences, University of Utah, Salt Lake City, UT 84112 USA; 2 Department of Ecology and Evolutionary Biology, Brown University, Providence, RI 02906 USA

**Keywords:** Biomechanics, Herpetology, Palaeontology

## Abstract

Most lizards walk and run with a sprawling gait in which the limbs are partly advanced by lateral undulation of the axial skeleton. Ribs and vertebrae are integral to this locomotor mode, but 3D motion of the axial skeleton has not been reported for lizard locomotion. Here, we use XROMM to quantify the relative motions of the vertebrae and ribs during slow treadmill locomotion in three savannah monitor lizards (*Varanus exanthematicus*) and three Argentine black and white tegus (*Salvator merianae*). To isolate locomotion, we selected strides with no concurrent lung ventilation. Rib rotations can be decomposed into bucket-handle rotation around a dorsoventral axis, pump-handle rotation around a mediolateral axis, and caliper rotations around a craniocaudal axis. During locomotion, every rib measured in both species rotated substantially around its costovertebral joint (8–17 degrees, summed across bucket, pump and caliper rotations). In all individuals from both species, the middle ribs rotated cranially through bucket and pump-handle motion during the propulsive phase of the ipsilateral forelimb. Axial kinematics during swing phase of the ipsilateral forelimb were mirror images of the propulsive phase. Although further work is needed to establish what causes these rib motions, active contraction of the hypaxial musculature may be at least partly responsible. Unilateral locomotor rib movements are remarkably similar to the bilateral pattern used for lung ventilation, suggesting a new hypothesis that rib motion during locomotion may have been an exaptation for the evolution of costal aspiration breathing in stem amniotes.

## Introduction

**Figure 1:**
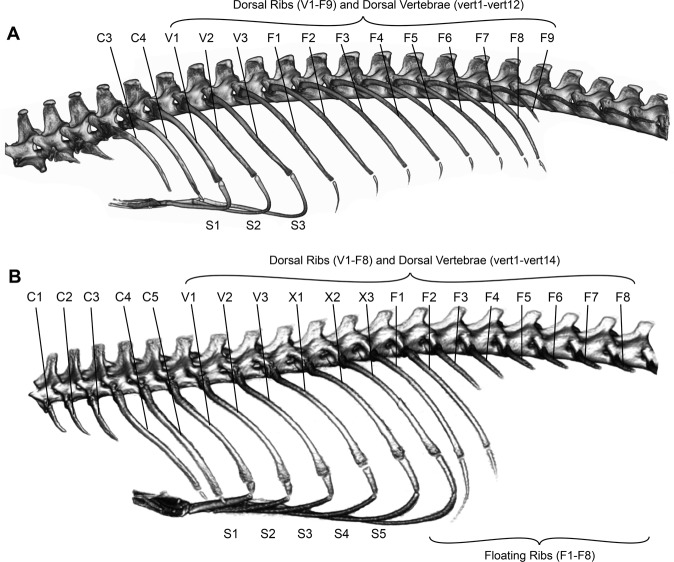
Axial anatomy of studied species. (**A**) Axial anatomy of *Varanus exanthematicus* and (**B**) *Salvator merianae* showing the naming scheme of vertebrae and ribs. C1-C5, cervical ribs 1–5; V1-V3, vertebral ribs 1–3; X1-X3, xiphisternal ribs 1–3; F1-F9, floating ribs 1–9; S1-S5; sternal ribs 1–5; vert1-vert14, dorsal (also sometimes called thoracic) vertebrae 1–14.

Stem tetrapods locomoted with a sprawling gait, in which the limbs were partly advanced by lateral undulation of the axial skeleton^1–3^. Modern lepidosaurs retain this locomotor mode^4–9^, but detailed 3D axial undulatory kinematics have not been measured in these animals. Motion of the axial skeleton and ribs has received considerable attention during lung ventilation^10–14^, but in what way do the vertebrae and ribs (Fig. 1) move during locomotion, if they move at all?

**Figure 2:**
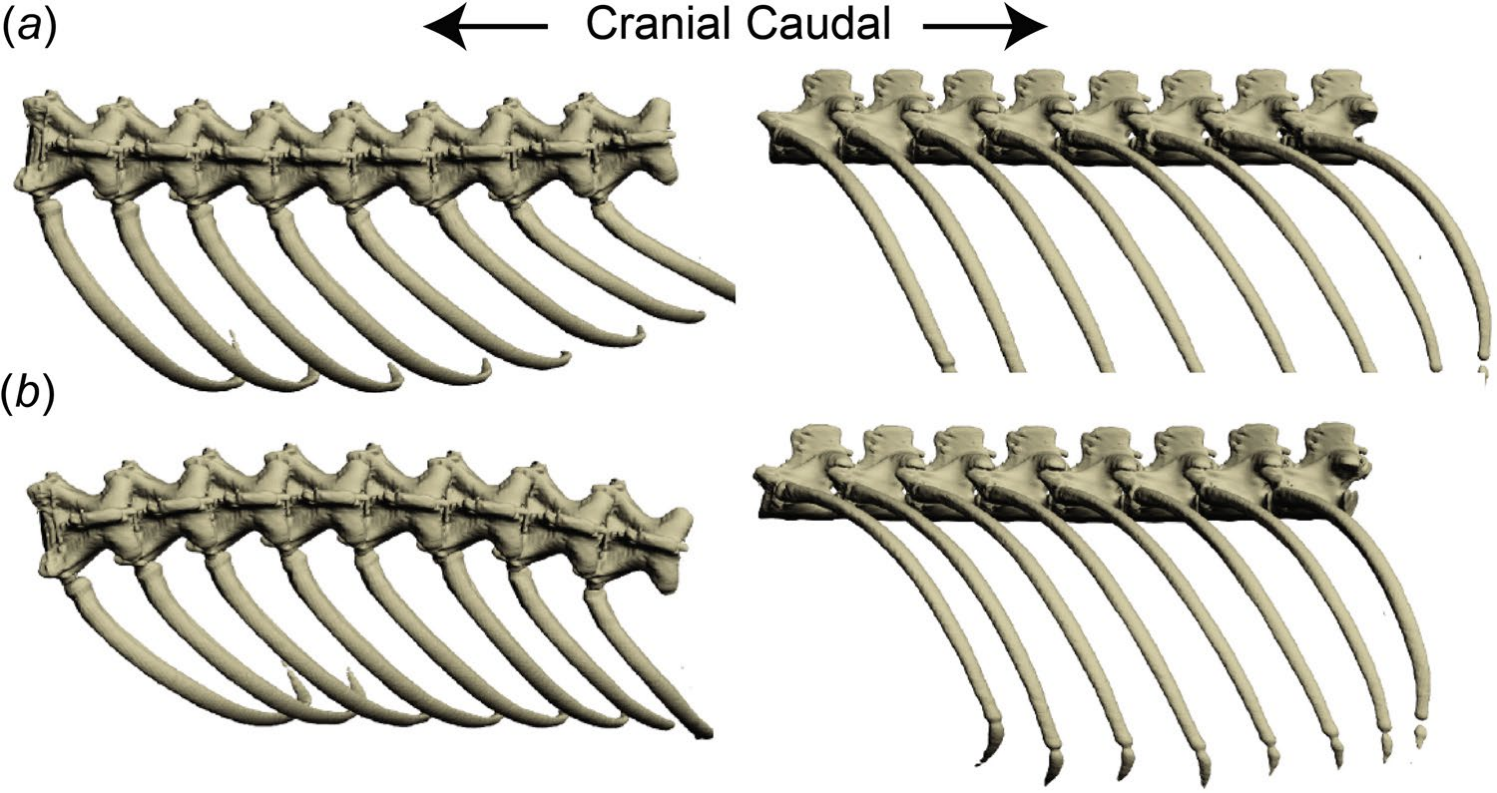
Null hypothesis of rib motion. (**a**) Dorsal (left) and lateral (right) views of the axial skeleton of *V. exanthematicus* with little vertebral column curvature (the resting CT scan pose of this individual). (**b**) Frame from null hypothesis (movie s3) showing lateral flexion at every intervertebral joint and no motion at the costovertebral joints. Vertebral rotations were simulated in Autodesk Maya with each intervertebral joint rotating 10 degrees with the associated ribs moving with (i.e. parented to) their corresponding vertebrae. Dorsal (left) and lateral (right) views of the axial skeleton at maximum concavity. Absence of motion at the costovertebral joints results in decrease in distance between the ribs, i.e. shortening of the intercostal spaces.

We expect intervertebral motions to be mainly restricted to lateral flexion about a dorsoventral axis because bony intervertebral processes largely restrict other motions in squamates^15^. There are many ways, however, that ribs might move during locomotion. Ribs might be pulled caudally due to hypaxial muscle contractions^5,16–18^, or cranially because of connections with the pectoral girdle^19^. Under a null costovertebral motion hypothesis, each rib would remain motionless relative to its corresponding vertebra and no rotations at the costovertebral joints would occur. A digital simulation of this null hypothesis shows that lateral flexion of the spine would alternatively bring the ribs closer together and farther apart, resulting in uneven intercostal spaces and crowding of the ribs (Fig. 2).

Costal kinematics are deceptively complex, especially in squamates because they have unicapitate ribs articulating with the vertebral column via shallow ball-and-socket joints that permit unrestrained rotation^15^. Three-dimensional rib rotations can be decomposed into three components, often defined relative to the animal’s body axes: bucket-handle rotation around a dorsoventral axis, caliper rotation around a craniocaudal axis, and pump-handle rotation around a mediolateral axis^10–14^. These motions can best be studied with X-ray Reconstruction of Moving Morphology (XROMM), which uses videofluoroscopy and computed tomography to reconstruct 3D skeletal shape and movement^20,21^.

Here, we used XROMM to measure the flexion of the vertebral column and the 3D rotations of ribs relative to their corresponding vertebrae (i.e. at the costovertebral joints) during slow treadmill locomotion in savannah monitor lizards and Argentine black and white tegus. We then compared these results from locomotion with costovertebral joint motions measured previously during lung ventilation (Cieri *et al*., 2018; Capano *et al*., 2019) in the same six individual lizards (three individuals from each species).

## Results

**Figure 3:**
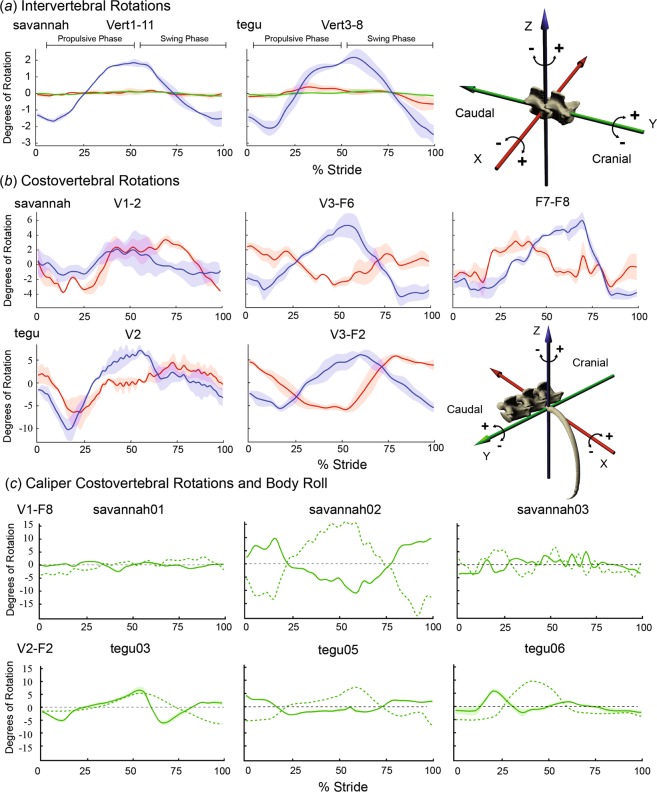
Intervertebral and costovertebral rotations during locomotion. (**a**) Mean intervertebral rotations at 10 intervertebral joints for all individuals of *V. exanthematicus* (savannah01–03) (left) and five intervertebral joints in *S. merianae* (tegu03,05,06) (center) during one locomotor stride. Blue line shows lateral bending at intervertebral joints; green and red are dorsoventral bending and torsion as shown by diagram of intervertebral JCS (right). (**b**) Bucket and pump-handle costovertebral rotations averaged across all individuals of *V. exanthematicus* (savannah) and *S. merianae* (tegu). Bottom right, diagram of the costovertebral JCS. Three distinct rotations are possible at the costovertebral joint: caliper motion about a craniocaudal Y-axis (green), bucket-handle rotation about a dorsoventral Z-axis (blue), and pump-handle rotation about a mediolateral X-axis (red). (**c**) Caliper costovertebral rotations (solid line) and vertebral column roll relative to the treadmill (dotted line) in each individual for *V. exanthematicus* (top), and *S. merianae* (bottom). For rotations in (**a–c**), stride durations were scaled to 100% and rotations were zeroed around their average. For (**a,b**), mean joint rotations were calculated among vertebrae or ribs within each individual and the mean taken across individuals (1 stride per individual) with the shaded area representing ±1 s.e. (n = 3 individuals). For (**c**), individuals differed from each other so mean joint rotations were calculated within each individual (over V1-F8 in *V. exanthematicus* and V3-F2 in *S. merianae*) with the shaded area representing ±1 s.e. See Fig. 1 for the numbering system for vertebrae and vertebral and floating ribs. Propulsive phase was approximately 0–50% of the stride and swing was 50–100%, as shown in (**a**).

Every rib measured in both species rotated substantially around its costovertebral joint during treadmill locomotion (Fig. 3). Mean rib rotation ranges (in degrees ± s.e.) summed across bucket, pump and caliper rotation for three *V. exanthematicus* and three *S. merianae* were: savannah01, 8.1 ± 3.3; savannah02, 14.8 ± 5.8; savannah03, 12.7 ± 3.6; tegu03, 13 ± 3.4; tegu05, 13.1 ± 4.9; tegu06, 16.8 ± 5.5. All ribs in both species bucketed cranially during the propulsive phase (i.e. stance phase) of the ipsilateral forelimb, and caudally during the swing phase of the ipsilateral forelimb (Fig. 3). At these walking speeds, the beginning of ipsilateral forelimb propulsive phase (forelimb footfall) roughly corresponds to the moment of maximum lateral convexity, and the beginning of ipsilateral forelimb swing phase corresponds to the moment of maximum lateral concavity (movie s1). In monitors, the magnitude of bucket-handle rotation was greater in the middle and posterior ribs (V3-F8) than in the anterior ribs (V1–2), but anterior and posterior ribs bucketed over similar ranges in tegus.

Pump-handle motions differed along the rib cage. In both monitors and tegus, the ventral tips of the anterior ribs (V1–2 in monitors, V2 in tegus) swung forward (cranially) with positive pump-handle motion during the ipsilateral propulsive phase and backward (caudally) during the swing phase (Fig. 3b). Pump-handle rotations among the middle ribs (V3-F6 in monitors, V3-F2 in tegus) were opposite in pattern, rotating backward during the propulsive phase and forward during the swing phase. In all three *V. exanthematicus*, F7 and F8 rotated forward during the propulsive phase and backward during the swing phase, similar to V1–2, although this motion was less pronounced in F7 than F8.

**Figure 4:**
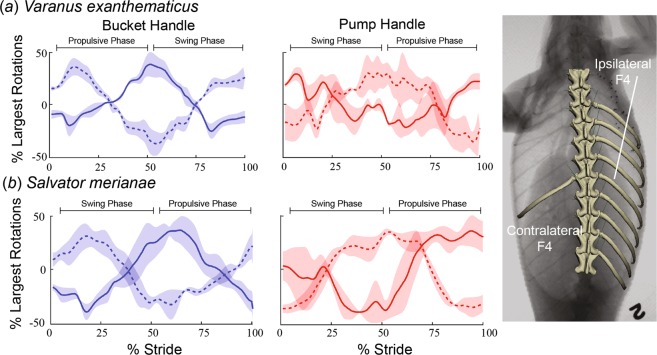
Kinematics of contralateral and ipsilateral rib are opposite during locomotion. (**a**) In *V. exanthematicus*, the bucket and pump-handle kinematics of the ipsilateral (solid) and contralateral (dashed) F4 rib are opposite. (**b**) In *S. merianae*, the bucket and pump-handle kinematics of the ipsilateral (solid) and contralateral (dashed) F1 rib are opposite. For each species, stride durations were scaled to 100% and rotations were zeroed around their average. Then mean joint rotations were calculated across individuals with the shaded area representing ±1 s.e. (n = 3 individuals). Right, diagram showing measured ipsilateral and contralateral ribs in *V. exanthematicus*. See Fig. 1 for the numbering system for vertebral and floating ribs.

Rib rotations on the other side of the body were opposite in polarity but of similar magnitude (Fig. 4), such that left-side and right-side bucket and pump motions during both propulsive and swing phases were opposite at any given moment.

Vertebrae rotated relative to each other over similar ranges (Fig. 3a) about a dorsoventral axis (i.e. lateral rotation of the vertebrae) in each individual. Little vertebral torsion, measured as the range of intervertebral rotations about a craniocaudal axis, or spinal flexion, measured as the range of intervertebral rotations about mediolateral axis, occurred in monitors or tegus (Fig. 3a).

Caliper motion of the vertebral and floating ribs was consistent within individuals, but differed among them (Fig. 3c). During the propulsive phase, the vertebral column of savannah02 rolled away from the striding forelimb, such that the spinous processes were tilted laterally towards the contralateral side of the trunk. The ribs of savannah02 calipered ventrally relative to their respective vertebrae during the propulsive phase, then dorsally during the swing phase. Little costal caliper motion or body roll occurred in savannah01 or savannah03. Caliper motion in the tegus was more consistent among individuals, with about five degrees of dorsal caliper during the ipsilateral propulsive phase. In both species, vertebral roll relative to the treadmill about a craniocaudal axis was typically coincident with and opposite in polarity to the magnitude and pattern of the roll of the vertebral column throughout the trunk (Fig. 3c).

## Discussion

**Figure 5:**
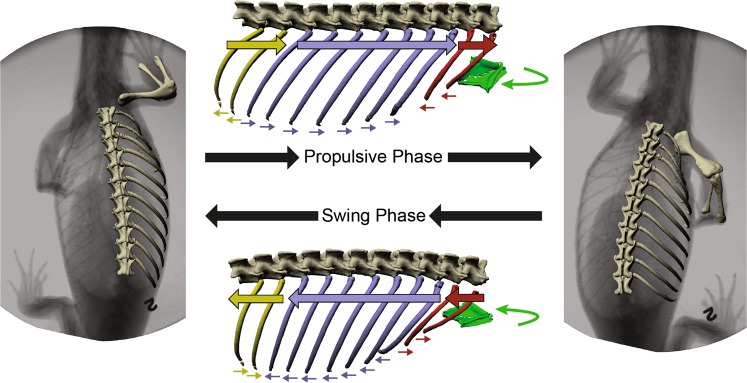
Synopsis of axial skeletal kinematics in *V. exanthematicus*. The ribs may be grouped into three regions based on similar rotation patterns: V1–2 (red), V3-F6 (purple), and F7–8 (yellow). Thick arrows superimposed close to the costovertebral joints represent rib motion due to bucket-handle rotation. Small, thin arrows located at the distal tips of the ribs represent the effect of pump-handle rotations at the tips. The rotations of the sternum (green) are shown by the curved green arrow adjacent to the sternum. Arrows indicate the rotations during the ipsilateral forelimb propulsive phase (top), and the ipsilateral swing phase (bottom). Motions measured in *S. merianae* were similar for V2 (red) and V3-F2 (purple).

We found clear evidence that ribs rotate substantially and similarly during locomotion in two distantly-related^22^ lizard species (Fig. 5). These findings lead us to reject the null hypothesis of little or no rotation at the costovertebral joints during locomotion in lizards (Fig. 2) and suggest that these locomotor rib kinematics may be basal to Squamata.

Prior electromyographic (EMG) work provides fairly strong evidence that the costal kinematics we observed are at least partially the result of active hypaxial muscle contraction, although they could instead be passive motions caused by the deformation of the trunk, or due to the competing influences of the extrinsic pectoral musculature and the muscles of lateral bending. Further work including EMG and measuring muscle fascicle length changes is clearly needed to identify the source of the observed kinematics, but previous investigations on similar species provide a basis for initial analysis. Work on *Iguana iguana* showed that the external intercostals and ventral portion of the internal intercostals are active during ipsilateral forelimb support^16^. These same muscles are active during the inspiratory phase of lung ventilation^23^, producing positive bucket-handle rotation^10^ that is similar to the positive bucket rotations we observed during the propulsive phase of locomotion (Figs. 3 and 5). Activity in the external intercostals and ventral portion of the internal intercostals of *Iguana iguana* during the propulsive phase, combined with the action of these muscles being positive bucket-handle rotation, suggests that the rib motions we observed during locomotion in *V. exanthematicus* and *S. merianae* were also produced by active hypaxial muscle contraction.

In addition, EMG work on the mangrove monitor (*Varanus salvator*) showed that rectus abdominis and obliquus externus are active while the ipsilateral trunk is bending towards concavity^5,18^. Axial muscle morphology is conserved in varanids^24,25^, so these muscles are almost certainly active in *V. exanthematicus* during trunk bending as well. On their own, contraction of these two muscles would pull the ribs caudally with negative bucket and positive pump-handle motions during the propulsive phase, the opposite pattern from what we found. Hence, it seems likely that other muscles, such as the intercostal muscles, contract simultaneously to produce the observed counter-rotations to these predicted forces.

It is also possible that the ribs are being moved cranially by locomotor actions of the pectoral muscles, several of which, i.e. (latissimus dorsi, pectoralis, and serratus anterior) insert on the anterior ribs, although the posterior aspect of the pectoralis indirectly connects with the ribs through the rectus abdominus^19,24^ and are active during late swing phase and most of the propulsive phase in *V. exanthematicus*^19^. Under this scenario, the pectoral musculature acting to move the forelimb and hypaxial muscles driving lateral bending would exert opposing actions on the ribs, and other hypaxial muscles would act to stabilize the rib cage against these actions and/or move the ribs. The fact that rib kinematics are similar between the middle and caudal ribs, which are not connected to the latissimus dorsi, serratus anterior, or directly to the pectoralis (Fig. 3b), suggests that forelimb musculature alone is not responsible for the reported rib kinematics. The extrinsic forelimb musculature might be responsible for the differences between the anterior and other ribs, however. The serratus anterior superficialis originates from the cervical and first thoracic (V1) rib and is active during the middle to late propulsive phase^19^, and as such would pull V1 dorsocranially towards its attachment on the scapula. The humerus and coracoid translate cranially relative to the sternum in order to increase step length during swing phase^19^ and muscular linkages between these bones and anterior ribs would pull them forwards, contributing to the negative pump-handle seen in these ribs near the end of the swing phase.

Notwithstanding the EMG evidence provided above, it is also possible that passive deformation of the trunk during the stride may be entirely responsible for, or at least contribute partially to rib rotations. In contrast to the rib crowding in the null model (Fig. 2 and Movie S3), similar width of the intercostal spaces was maintained along the ribcage in the walking lizards (Movies 1–2. Thus it is possible, although we consider it unlikely given the EMG studies described above, that the ribs rotated entirely passively to maintain equal intercostal spacing during lateral bending by the resistance to compression of the intercostal tissues. Instead, some combination of active and passive rotation at the costovertebral joints seems quite plausible. The biomechanics of the trunk during locomotion are quite complex, and additional work is needed to confidently establish the relationships between axial and appendicular muscles and skeletal kinematics. Our results nonetheless lead to several interesting, though speculative, hypotheses about the locomotor-ventilatory constraint and the initial evolution of costal breathing.

Our goal in this study was to measure rib kinematics during slow locomotion, and not a combination of lung ventilation and locomotion at the same time (costal ventilation during locomotion at slow speeds can occur in lizards)^17,26–28^. Hence, we carefully selected locomotor cycles in which the X-ray images showed no evidence of lung volume change. The results provide some evidence that we were successful: rib rotations on the side of the body contralateral to forelimb support were similar in magnitude and opposite in polarity during the same stride (Fig. 4). If the ribs were rotating bilaterally to produce lung ventilation in the same stride, then the ventilatory motions would add to the magnitude of one side and subtract from the other. Varanids^28^, but not teiids^29,30^, gular pump during exercise, so we also rejected any locomotor strides in which changes in gular cavity volume were visible in the X-ray movies. Breaths or gular pumps small enough to be unnoticed in the trial selection process would also be too small to significantly affect our kinematic results. Most costal locomotor breaths are of low tidal volume and are not thought to contribute significantly to ventilation^17,26,27^.

The ipsilateral and contralateral rib motions reported here (Figs. 3 and 4) lend support to the locomotor-ventilatory constraint hypothesis in lizards^1,17^. These results suggest that ordinary costal breaths during lateral undulation may be impeded as the left and right sets of ribs are engaged in opposite motions while the animals are locomoting. Our locomotor results are from slow treadmill locomotion, and not the high-speed locomotion that is hypothesized to seriously compromise the ability of lizards to breathe while running^1,17,28^. Nonetheless, these results show that the ribs actually move during locomotion - they are not simply stabilized in place, and that the movements occur unilaterally, findings that are not consistent with the bilateral rib motions that produce lung ventilation in lizards.

**Figure 6:**
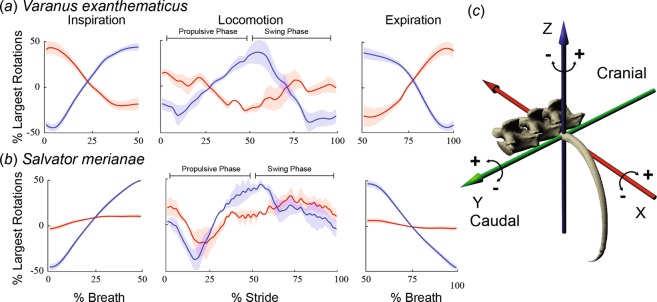
Similarity of costovertebral kinematics during ventilation and locomotion. For *V. exanthematicus* (**a**), mean bucket and pump-handle rotations at the costovertebral joints of the third true and first through sixth floating ribs (locomotion) and the third true as well as the second fourth and sixth floating ribs (ventilation). For *S. merianae* (**b**), mean bucket and pump-handle rotations at the costovertebral joints of the second true rib (locomotion and ventilation). For each species, stride and breath durations were scaled to 100% and rotations were zeroed around their average and scaled to bucket-handle range. Then mean joint rotations were calculated within each individual and the mean taken across individuals (1 stride and breath per individual) with the shaded area representing ±1 s.e. (n = 3 individuals). Rib kinematics during ventilation from^11^ and^12^. For ventilation, multiple ribs were averaged into a single curve for each species and separated into expiration and inspiration to facilitate comparisons between locomotion and ventilation as breaths normally begin with expiration in both species.

On their own, however, the unilateral bucket and pump rotations reported here during locomotion are similar to the bilateral rotations found during lung ventilation from prior studies on the same individual lizards (Fig. 6). Caliper rotation contributes little to lung ventilation in these species^11,12^, and was variable among individuals during locomotion (Fig. 2c), so will not be considered further. In both species, the observed bucket and pump rotations during the propulsive phase are similar to those during inspiration, and rotations during the swing phase are similar to those during expiration (Fig. 6). During inspiration, the middle ribs of monitors (V3-F6) were found to bucket cranially while pump-handle motion swung them caudally^11^, the same pattern found during ipsilateral front limb support. The second tegu true rib (V2) bucketed and pumped cranially during the second half of the ipsilateral propulsive phase and caudally during swing phase in a similar pattern to inspiration and expiration, respectively, in this species^12^. Ribs caudal to V3 do not contribute substantially to ventilation in *S. merianae* but share the same locomotor pattern as *V. exanthematicus* (Fig. 3b).

Finally, the fact that the ribs move at all during locomotion, and the similarity between the reported rib kinematics during ventilation and locomotor kinematics in both species (Fig. 6) suggest an intriguing hypothetical intermediate stage in the evolution of costal aspiration breathing in amniotes. The primitive condition for tetrapods is buccal pump ventilation, where air is forced into the lungs by the musculature of the head. Costal aspiration breathing most likely evolved in stem amniotes^29,31^ and represents a shift from the musculoskeletal system of the head being responsible for lung ventilation to the musculoskeletal system of the trunk taking over the responsibility for ventilation. An intermediate step in this transformation is the use of trunk muscles to power expiration while retaining the buccal pump for inspiration, as seen in extant amphibians^32–34^. This expiration pump does not necessarily involve the ribs, as simply surrounding the body cavity with appropriately-oriented musculature, such as the transverse abdominis, and contracting that musculature will squeeze air out of the lungs.

How the ribs and intercostal musculature were recruited for inspiration has been a more challenging evolutionary transformation to reconstruct^35^. Based on the locomotor rib kinematics reported here, we propose the following hypothetical evolutionary scenario: 1) robust ribs with mobile costovertebral joints were present in early tetrapods; 2) sprawling, lateral undulatory locomotion evolved in early tetrapods, before the evolution of costal aspiration breathing; 3) unilateral rib rotations associated with a sprawling gait and lateral undulatory locomotion happened to be rotations that, if expressed bilaterally, would expand the thorax and pull air down into the lungs; 4) inspiration by aspiration evolved in stem amniotes when the rib kinematics of the propulsive phase were expressed bilaterally.

In this scenario, rib rotations, or the increased magnitude of rib rotations, during locomotion was an exaptation that evolved under selection for locomotion and subsequently co-opted and modified for lung ventilation. Rib motion during locomotion may have been advantageous not only for avoiding rib crowding, but also for balancing the competing influences of the extrinsic pectoral and hypaxial lateral bending musculature on the rib cage and also increasing the magnitude or efficacy of lateral bending powered by hypaxial muscle contraction. Because the hypaxial musculature that causes lateral bending attaches onto the ribs^5,16^ it is possible that some active rib motion is necessary to stabilize the ribs at the costovertebral joint – otherwise contraction of the hypaxial muscles would only collapse the rib cage instead of contributing to lateral spinal flexion. Cranial rib rotation might even increase the amount of lateral bending caused by a given amount of hypaxial muscle contraction by increasing the dynamic distance between the origins and attachments of the external oblique and rectus abdominus.

One objection to this exaptation hypothesis could be that the locomotor rib kinematics measured here for two species of lizards may not be ancestral for amniotes. The fact that the two lizard species in this study are distantly-related within squamates^22^, however, suggests that they are ancestral. It is speculative whether these rib kinematics would also have been ancestral for amniotes, but lizards have the most similar body form to early amniotes and are often used as analogs e.g.^36^. Finally, even if the reported rib kinematics are later shown to be passive in origin or due to the competing actions of the extrinsic pectoral and lateral-bending hypaxial musculature, the resulting rib motility during locomotion could have set the stage for active rib motions over a greater range during ventilation.

Another objection to this scenario could be that the ribs of early amniotes were two-headed, unlike the single-headed ribs of lizards, and these bicapitate ribs in amniotes would have constrained rib motions to hinge-like rotation about an axis connecting the two rib heads. In contrast, costovertebral joints of lizards are more similar to ball-and-socket joints that permit a much wider range of rotations, which makes it more remarkable that the unilateral locomotor kinematics are so consistently similar to the bilateral ventilatory kinematics (Fig. 6). We hypothesize that the key to this putative exaptation was that the axis of rotation of the ribs of early amniotes, constrained by bicapitate costovertebral joints that were perhaps shaped by selection for locomotion^12,37^, happened to be a rotation that would expand the thorax and pull air down into the lungs, if expressed bilaterally.

## Materials and Methods

Skeletal kinematics during slow treadmill locomotion were collected from three savannah monitor lizards, *Varanus exanthematicus*, (savannah01,1.4 kg; savannah02, 1.2 kg; savannah03, 0.95 kg) and three Argentine black and white tegus, *Salvator merianae*, (tegu03, 1.2 kg; tegu05, 2.1 kg; tegu06, 1.5 kg). All procedures were approved by the Institutional Animal Care and Use Committee (IACUC) of Brown University and followed guidelines and policies set forth by the IACUC of Brown University. X-ray and CT scan data presented in this study are available at (www.xmaportal.org) in study IDs BROWN42 and BROWN38. Video data are stored with their essential metadata in accordance with best practices for video data management in organismal biology^38^.

**Figure 7:**
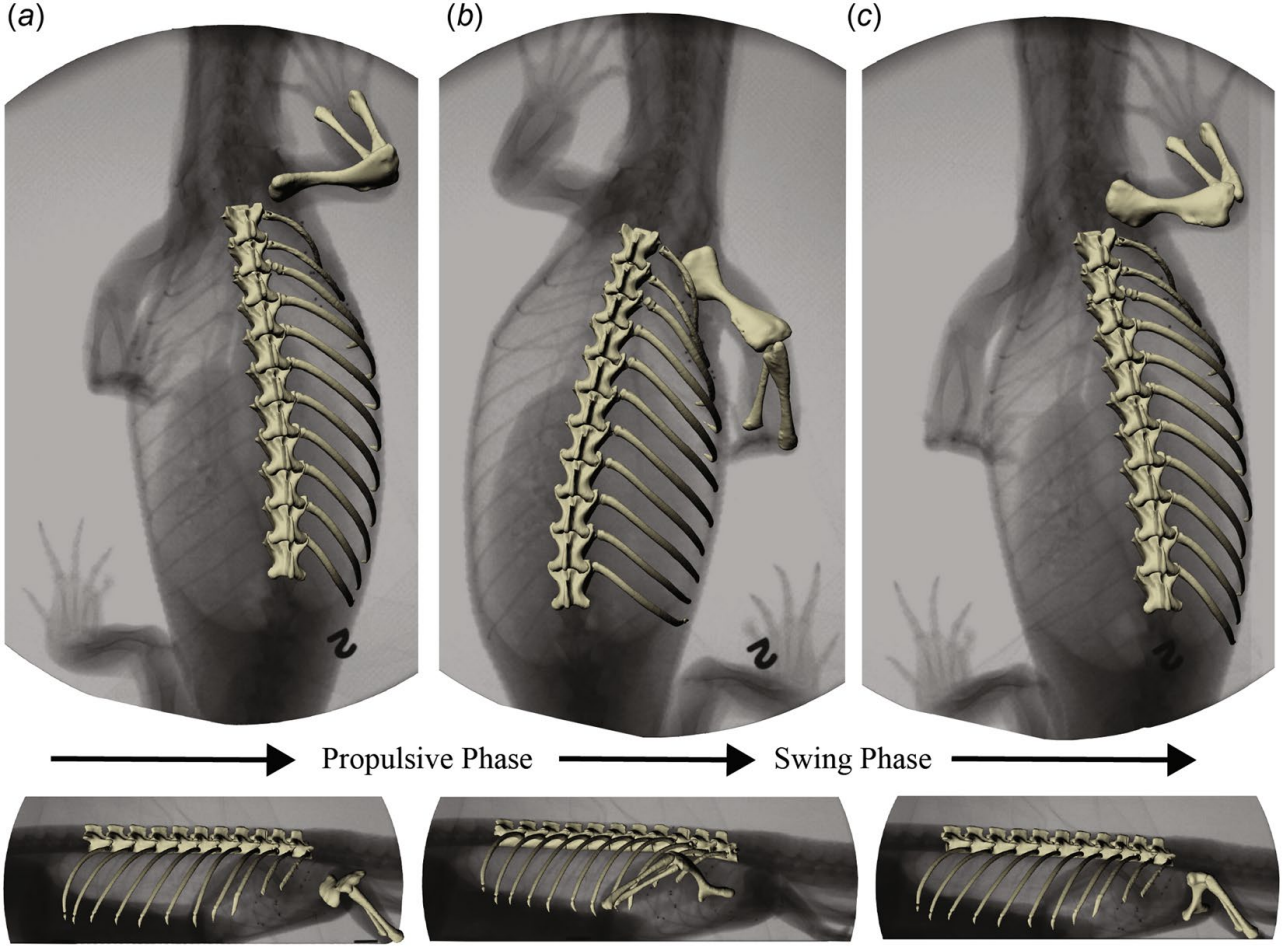
Definition of locomotor stride. XROMM animations were created over the full course of a locomotor stride in both species (*V. exanthematicus* depicted only). Dorsal view (top) and lateral view (bottom) depicting the axial elements, as well as the ipsilateral humerus, radius, and ulna superimposed on the corresponding X-rays. Axial kinematic data were collected for one full cycle starting and ending when the right front limb contacts the treadmill (**a**). The propulsive phase continues until the limb breaks contact with the treadmill (**b**), starting the swing phase which continues until the next footfall (**c**). Stride durations were 1.85 seconds (sav01), 1.56 seconds (sav02), 1.5 seconds (sav03), 1.5 seconds (teg03), 2.83 (te05), and 3.25 seconds (tegu06).

Animations were generated with marker-based^20^ and markerless Scientific Rotoscoping^21^ XROMM. Details of marker implantation for these lizards may be found in the corresponding papers on lung ventilation^11,12^. The goal of this study was to analyze locomotion without simultaneos lung ventilation, so we carefully selected locomotor cycles in which the X-ray images showed no evidence of volume change in the lungs or gular cavity (see Fig. 7 for how clearly the lungs and gular cavity can be seen in the X-ray images). Markers were tracked with XMALab software^39^, and marker tracking precison for these marker sets was reported in the lung ventilation studies^11,12^. In the savannah monitors, one dorsal vertebra in each individual (savannah01, vert2; savannah02, vert3; savannah03, vert1) as well as vertebral ribs V1–2 were animated with marker-based XROMM (see Fig. 1 for rib and vertebra naming conventions). Other vertebrae and ribs were hierarchically parented to the marker-based vertebrae for Scientific Rotoscoping using Autodesk Maya animation software (Autodesk Inc., San Rafael, CA, USA). Intervertebral rotations were manually aligned, and translation was constrained by the marked vertebrae. Translation of ipsilateral V3 and floating ribs F1–8 as well as the contralateral F4 were controlled by vertebral translation while rotations were aligned manually. Scientific Rotoscoping of ribs in this dataset was previously validated against marker-animated ribs^11^. For the tegus, one dorsal vertebra in each individual (tegu03, vert3; tegu05, vert1; tegu06, vert3) as well vertebral rib V2 (all individuals) were animated with marker-based XROMM. As in the monitor lizards, other vertebrae and ribs were hierarchically parented to the marker-based vertebrae in Autodesk Maya. Intervertebral rotations were manually aligned, and translation was constrained by the marked vertebrae. Translation of ipsilateral V3, xiphisternal ribs X1–3 and floating ribs F1–2 as well as ipsilateral F1were controlled by vertebral translation while rotations were aligned manually.

Due to the extensive time and effort required for scientific rotoscoping of the whole thorax, only one stride was animated for each animal (Fig. 7). In the monitors, for example, the animations each have twenty handles that were individually manipulated to generate motions, resulting in a total of 981 manual registrations of the pose the bones relative to the video images (280, 287, and 414 each for savannah01–03). One stride per individual is an unusually small sample size for locomotion studies, but the main features of the vertebral and costal kinematics, and particularly the direction of rib rotation during the stance and propulsive phases, did not vary among individuals or species. We base our conclusions in this study on these invariant components of the kinematic traces. Substantial differences in axial kinematics were not observed during an initial, non-quantitative examination of a larger sample size so we are relatively confident that the strides reported here are representative of the axial skeletal kinematics of these animals under these recording conditions and a larger sample size of analyzed strides would support the same conclusions.

Relative motions between bones were measured using joint coordinate systems (JCS) with polarity determined by the right-hand rule and a Euler angle rotation order of ZYX. The zero position for intervertebral JCSs was defined with the vertebral column aligned in coronal and sagittal planes (Fig. 3a). The zero-position for costovertebral JCSs was defined where the vertebral column was straight along a sagittal plane and the dorsal portions of the ribs were aligned along a coronal plane (Fig. 3b). Another JCS (not figured) measured roll of the vertebral column about a craniocaudal axis relative to the treadmill surface. The zero position for this JCS was established with vertebrae parallel to the treadmill along the coronal and sagittal planes.

## Supplementary information

Supplementary information.

Supplementary Movie S1.

Supplementary Movie S2.

Supplementary Movie S3.

Supplementary Tables
