# Supplementary material for: Locomotor rib kinematics in two species of lizards and a new hypothesis for the evolution of aspiration breathing in amniotes
Source: Sci Rep. 2020 May 12;10:7739. doi: 10.1038/s41598-020-64140-y (PMC7217971; doi:10.1038/s41598-020-64140-y)
Supplement: Supplementary file 1 — Supplementary information. [file 41598_2020_64140_MOESM1_ESM.docx]

**Supplementary Material**

Locomotor rib kinematics in two species of lizards and a new hypothesis for the evolution of aspiration breathing in amniotes

Robert L. Cieri^1^*^†^, Samuel T. Hatch^1†^, John G. Capano^2^, and Elizabeth L. Brainerd^2^

1: School of Biological Sciences, University of Utah, Salt Lake City, UT 84112, USA

2: Department of Ecology and Evolutionary Biology, Brown University, Providence, RI 02906, USA

*Author for correspondence ([bob.cieri@gmail.com](mailto:bob.cieri@gmail.com))

247 S 1400 E

201 South Biology

The University of Utah

Salt Lake City, UT 84112

†These two authors contributed equally and are listed in alphabetical order

**Supplementary Movie 1:**Biplanar X-ray videos depicting a complete locomotor stride in *V. exanthematicus*. Dorsal (top), and lateral view (bottom).

**Supplementary Movie 2:**XROMM animation overlaid on biplanar X-ray videos. Dorsal (top) and lateral (bottom) views show the animated sternum, vertebra (vert1-11), and ribs (V1-F8) over a complete locomotor stride in *V. exanthematicus.*

**Supplementary Movie 3:**Video depicting simulated axial motions of the null model based on *V. exanthematicus* assuming lateral undulation without costovertebral rotations. The simulated intervertebral joint rotations are exaggerated relative to measured *in vivo* motions (10 deg versus about 2 deg) to emphasize the effect of zero costovertebral rotation.

**Supplementary Table 1:** Intervertebral rotation ranges around a dorsoventral axis expressed in raw Euler angles (degrees).

**Supplementary Table 2:** Rotations in degrees of vertebral ribs V1-3 at the costovertebral joints for savannah01, savannah02, and savannah03.

**Supplementary Table 3:** Rotations in degrees of vertebral ribs V2-3 at the costovertebral joints for tegu03, tegu05, and tegu06.

**Supplementary Table 4:** Rotations in degrees of floating ribs F1-4 at the costovertebral joints for savannah01, savannah02, and savannah03.

**Supplementary Table 5:** Rotations in degrees of floating ribs F5-8 at the costovertebral joints for savannah01, savannah02, and savannah03.

**Supplementary Table 6:** Rotations in degrees of floating ribs F1-5 at the costovertebral joints for tegu03, tegu05, and tegu06.
