# Supplementary material for: Locomotor rib kinematics in two species of lizards and a new hypothesis for the evolution of aspiration breathing in amniotes
Source: Sci Rep. 2020 May 12;10:7739. doi: 10.1038/s41598-020-64140-y (PMC7217971; doi:10.1038/s41598-020-64140-y)
Supplement: Supplementary file 5 — Supplementary Tables [file 41598_2020_64140_MOESM5_ESM.pdf]

Table S1: Intervertebral rotation ranges around a dorsoventral axis expressed in raw Euler angles (degrees).

	**savannah01**	**savannah02**	**savannah03**	**tegu03**	**tegu05**	**tegu06**
vert1-2	4.5	0	7.5	NA	NA	NA
vert2-3	6.1	7.6	11.2	NA	NA	NA
vert3-4	11.7	9.6	5.5	7.1	8.5	8.2
vert4-5	5.3	5.8	6.7	8.3	8.3	9.6
vert5-6	6.7	9.6	7.8	5.6	6.3	7.5
vert6-7	7	9.6	7.4	7.3	10.3	4.3
vert7-8	5.8	7.1	9.2	5.8	7.6	0.0
vert8-9	7.8	9.1	12.9	NA	NA	NA
vert9-10	8.3	8.8	10.3	NA	NA	NA
vert10-11	11.9	9.6	6.6	NA	NA	NA

Table S2: Rotations in degrees of vertebral ribs V1-3 at the costovertebral joints for savannah01, savannah02, and savannah03.

	**V1**			**V2**			**V3**		
	**sav01**	**sav02**	**sav03**	**sav01**	**sav02**	**sav03**	**sav01**	**sav02**	**sav03**
**Pump Handle (X axis)**[1]									
max	1.00	8.37	10.25	-23.17	-6.45	8.69	-16.89	-0.99	7.31
min	-10.95	-3.83	3.45	-31.71	-17.73	-0.26	-29.94	-13.74	-7.55
magnitude[2]	11.95	12.20	6.80	8.54	11.28	8.95	13.05	12.75	14.86
**Caliper Motion (Y axis)**[1]									
max	-4.02	1.03	-6.98	-0.94	3.33	-11.27	-5.73	8.76	-4.66
min	-12.37	-19.19	-15.28	-5.66	-18.65	-19.52	-11.20	-23.09	-16.64
magnitude[2]	8.35	20.21	8.30	4.71	21.99	8.25	5.47	31.85	11.98
**Bucket Handle (Z axis)**[1]									
max	-22.09	-17.33	2.64	-31.30	-17.93	1.67	-37.09	-14.53	-4.61
min	-29.22	-23.78	-5.53	-37.68	-24.67	-14.72	-48.03	-31.14	-17.88
magnitude[2]	7.13	6.45	8.17	6.38	6.74	16.39	10.94	16.61	13.28

[1]Axes of rotation are illustrated in figure 3

[2]Magnitude is defined as the difference in degrees between the maximum and minimum as seen in the table

Table S3: Rotations in degrees of vertebral ribs V2-3 at the costovertebral joints for tegu03, tegu05, and tegu06.

	V2			V3		
	**teg03**	**tegu05**	**teg06**	**teg03**	**tegu05**	**teg06**
**Pump Handle (X axis)[1]**						
max	-26.32	5.39	9.74	-9.00	10.38	-8.40
min	-35.25	-2.00	-12.90	-22.05	-1.49	-28.55
magnitude[2]		8.93	7.38	22.63	13.05	11.87
**Caliper Motion (Y axis)[1]**						
max	9.39	1.29	12.97	12.94	1.18	12.38
min	3.20	-8.41	-2.15	-1.63	-7.21	-2.27
magnitude[2]	6.19	9.70	15.11	14.57	8.40	14.66
**Bucket Handle (Z axis)[1]**						
max	-6.53	7.73	0.73	6.83	-18.67	4.68
min	-18.76	-10.69	-24.18	-9.80	-36.67	-14.36
magnitude[2]	12.23	18.43	24.91	16.63	18.00	19.04

Table S4: Rotations in degrees of floating ribs F1-4 at the costovertebral joints for savannah01, savannah02, and savannah03.

	F1			F2			F3			F4		
	**sav01**	**sav02**	**sav03**	**sav01**	**sav02**	**sav03**	**sav01**	**sav02**	**sav03**	**sav01**	**sav02**	**sav03**
**Pump Handle (X axis)[1]**												
max	-15.69	-0.13	8.17	-19.76	-3.89	0.46	-25.60	-3.99	-3.16	-26.51	-7.77	-4.13
min	-22.26	-9.78	-11.41	-31.79	-16.43	-9.36	-39.28	-14.34	-15.27	-35.74	-19.91	-14.79
magnitude[2]	6.57	9.65	19.58	12.03	12.54	9.82	13.67	10.34	12.12	9.22	12.14	10.66
**Caliper Motion (Y axis)[1]**												
max	-7.60	7.69	-4.52	-7.07	12.16	2.60	-4.68	11.49	7.37	-1.63	10.49	9.19
min	-11.86	-13.36	-14.55	-12.44	-10.56	-6.02	-7.82	-9.42	-2.90	-5.67	-9.20	-1.85
magnitude[2]	4.27	21.05	10.03	5.36	22.72	8.62	3.14	20.91	10.27	4.04	19.69	11.05
**Bucket Handle (Z axis)[1]**												
max	-31.30	-19.46	-5.97	-32.90	-18.24	-8.77	-33.63	-18.30	-12.87	-26.72	-13.83	-11.50
min	-38.42	-26.48	-18.39	-37.77	-29.72	-24.41	-39.81	-30.88	-30.76	-34.06	-28.81	-32.33
magnitude[2]	7.12	7.02	12.42	4.87	11.49	15.64	6.17	12.58	17.88	7.34	14.97	20.82

[1]Axes of rotation are illustrated in figure 3

[2]Magnitude is defined as the difference in degrees between the maximum and minimum as seen in the table

Table S5: Rotations in degrees of floating ribs F5-8 at the costovertebral joints for savannah01, savannah02, and savannah03.

	F5			F6			F7			F8		
	**sav01**	**sav02**	**sav03**	**sav01**	**sav02**	**sav03**	**sav01**	**sav02**	**sav03**	**sav01**	**sav02**	**sav03**
**Pump Handle (X axis)[1]**												
max	-22.33	-7.71	-2.74	-16.64	-6.91	5.37	-9.08	-9.76	7.99	3.33	-8.52	10.19
min	-32.05	-16.54	-10.96	-24.53	-13.73	-6.61	-12.35	-20.18	-8.20	-11.03	-22.32	-4.55
magnitude[2]	9.72	8.83	8.22	7.90	6.82	11.99	3.27	10.42	16.19	14.36	13.80	14.74
**Caliper Motion (Y axis)[1]**												
max	-0.34	11.52	11.99	0.02	9.55	9.65	8.20	8.70	6.47	11.87	14.98	5.87
min	-5.08	-8.35	-0.42	-5.72	-10.74	-2.92	2.12	-11.12	-5.49	1.44	-5.51	-5.04
magnitude[2]	4.73	19.87	12.41	5.74	20.30	12.58	6.09	19.82	11.96	10.42	20.48	10.90
**Bucket Handle (Z axis)[1]**												
max	-20.90	-14.98	-8.56	-20.71	-10.86	-10.95	-8.12	-11.12	-8.50	-7.40	-10.90	-5.70
min	-33.75	-27.49	-28.22	-31.03	-24.62	-24.06	-19.11	-22.64	-22.18	-19.31	-24.61	-22.17
magnitude[2]	12.85	12.51	19.66	10.32	13.76	13.12	11.00	11.52	13.68	11.91	13.71	16.47

[1]Axes of rotation are illustrated in figure 3

[2]Magnitude is defined as the difference in degrees between the maximum and minimum as seen in the table

Table S6: Rotations in degrees of floating ribs F1-5 at the costovertebral joints for tegu03, tegu05, and tegu06.

	X1			X2			X3			F1			F2		
	**teg03**	**teg05**	**teg06**	**teg03**	**teg05**	**teg06**	**teg03**	**teg05**	**teg06**	**teg03**	**teg05**	**teg06**	**teg03**	**teg05**	**teg06**
**Pump Handle (X axis)[1]**															
max	-7.71	9.49	-7.39	-6.10	9.80	-8.18	-8.56	-2.05	-12.49	-6.44	4.13	-18.25	-2.17	8.90	-17.16
min	-24.33	-2.83	-35.20	-26.70	-7.96	-32.10	-22.39	-23.13	-30.19	-20.85	-11.57	-34.56	-15.71	-5.69	-36.06
magnitude[2]	16.63	12.32	27.81	20.60	17.77	23.92	13.84	21.08	17.70	14.40	15.70	16.32	13.54	14.59	18.90
**Caliper Motion (Y axis)[1]**															
max	7.40	-0.93	8.53	8.46	-4.90	2.83	4.87	-3.32	-2.03	3.24	-6.06	-1.15	-8.58	0.99	-1.63
min	-5.55	-8.84	-6.08	-9.89	-12.79	-7.69	-11.33	-11.43	-7.67	-11.71	-12.54	-9.27	-16.40	-5.81	-17.82
magnitude[2]	12.95	7.91	14.61	18.35	7.89	10.51	16.19	8.11	5.64	14.95	6.48	8.13	7.82	6.80	16.19
**Bucket Handle (Z axis)[1]**															
max	3.56	-18.61	3.73	7.20	-19.81	3.91	9.45	-26.36	5.97	12.16	-19.44	9.49	13.13	-10.62	12.31
min	-6.86	-39.58	-12.88	-6.15	-37.70	-8.28	-2.78	-38.84	-5.27	-4.08	-33.10	-6.92	-2.63	-28.26	-7.29
magnitude[2]	10.42	20.98	16.61	13.35	17.89	12.20	12.23	12.48	11.24	16.25	13.67	16.41	15.76	17.64	19.59
